# Radiotracer Innovations in Breast Cancer Imaging: A Review of Recent Progress

**DOI:** 10.3390/diagnostics14171943

**Published:** 2024-09-03

**Authors:** Mohamad Haidar, Joe Rizkallah, Omar El Sardouk, Nour El Ghawi, Nadine Omran, Zeinab Hammoud, Nina Saliba, Arafat Tfayli, Hiba Moukadem, Ghina Berjawi, Lara Nassar, Fahad Marafi, Partha Choudhary, Habibollah Dadgar, Alyaa Sadeq, Alain S. Abi-Ghanem

**Affiliations:** 1 Department of Diagnostic Radiology, American University of Beirut, Beirut 1107-2020, Lebanon; jr56@aub.edu.lb (J.R.); oe34@aub.edu.lb (O.E.S.); ne130@aub.edu.lb (N.E.G.); no15@aub.edu.lb (N.O.); zh87@aub.edu.lb (Z.H.); ns204@aub.edu.lb (N.S.); gb02@aub.edu.lb (G.B.); ln07@aub.edu.lb (L.N.); 2 Division of Hematology and Oncology, Department of Internal Medicine, American University of Beirut, Beirut 1107-2020, Lebanon; at35@aub.edu.lb (A.T.); hm127@aub.edu.lb (H.M.); 3 Jaber Al-Ahmad Centre for Molecular Imaging, Kuwait City 70031, Kuwait; fahadmarafi@yahoo.com (F.M.); alyaa.kbnm@gmail.com (A.S.); 4 Department of Nuclear Medicine, Rajiv Gandhi Cancer Institute and Research Centre, New Delhi 110085, India; pschoudhary@hotmail.com; 5 Cancer Research Center, RAZAVI Hospital, Imam Reza International University, Mashhad 9198613636, Iran; reza.lt.dadgar@gmail.com; 6 Department of Diagnostic Radiology, American University of Beirut Medical Center, Beirut 1107-2020, Lebanon; aa277@aub.edu.lb

**Keywords:** breast cancer, molecular imaging, nuclear medicine, positron emission tomography, radiopharmaceuticals, radiotracers, tumor detection, ^18^F-fluorodeoxyglucose

## Abstract

This review focuses on the pivotal role of radiotracers in breast cancer imaging, emphasizing their importance in accurate detection, staging, and treatment monitoring. Radiotracers, labeled with radioactive isotopes, are integral to various nuclear imaging techniques, including positron emission tomography (PET) and positron emission mammography (PEM). The most widely used radiotracer in breast cancer imaging is ^18^F-fluorodeoxyglucose (^18^F-FDG), which highlights areas of increased glucose metabolism, a hallmark of many cancer cells. This allows for the identification of primary tumors and metastatic sites and the assessment of tumor response to therapy. In addition to ^18^F-FDG, this review will explore newer radiotracers targeting specific receptors, such as estrogen receptors or HER2, which offer more personalized imaging options. These tracers provide valuable insights into the molecular characteristics of tumors, aiding in tailored treatment strategies. By integrating radiotracers into breast cancer management, clinicians can enhance early disease detection, monitor therapeutic efficacy, and guide interventions, ultimately improving patient outcomes. Ongoing research aimed at developing more specific and sensitive tracers will also be highlighted, underscoring their potential to advance precision medicine in breast cancer care.

## 1. Introduction

Breast cancer is the most frequently diagnosed cancer in the world, making it a serious public health concern. Age is the most significant risk factor, with older women experiencing the highest incidence rates. Concerning breast cancer types, most cancers are adenocarcinomas, with 85% developing in the breast ducts and 15% in the lobular epithelium [1,2]. Early detection of breast cancer is crucial for improving survival rates. Mammography significantly reduces breast cancer-related deaths, but dense breast tissue can make detection challenging. Advanced imaging methods like magnetic resonance imaging (MRI), contrast-enhanced mammography, and molecular breast imaging are more sensitive in dense breasts. MRI or ultrasound are used more frequently for individuals with hard-to-image breasts or at high risk, such as women with BRCA1 or BRCA2 mutations [3]. Nevertheless, computed tomography (CT) scans and MRIs do not always provide conclusive results for many individuals because they only show structural and anatomical changes at the main cancer site and distant metastases. On the other hand, molecular imaging techniques are more effective in staging and detecting treatment response and recurrence, particularly when structural changes caused by treatment or surgery are present. Positron emission tomography/computed tomography (PET/CT) using ^18^F-fluorodeoxyglucose (^18^F-FDG) is especially useful for risk stratification, staging, and predicting responses to neoadjuvant chemotherapy (NAC), but with limited sensitivity and specificity in some subtypes of breast cancer [4,5]. In addition to ^18^F-FDG, many other radiotracers have been developed to complement its diagnostic capabilities. This review aims to elaborate on the role of molecular imaging in the diagnosis of breast cancer and highlight both current and emerging radiotracers and techniques in nuclear imaging of breast cancer.

## 2. Sources and Selection Criteria

In order to compile the relevant literature for this review on the use of radiotracers in breast cancer imaging, we conducted a comprehensive search using multiple academic databases such as PubMed and Medline. The search strategy included a combination of keywords such as, but not limited to, ‘radiotracers’, ‘breast cancer’, ‘positron emission tomography (PET)’, ‘nuclear medicine’, and ‘molecular imaging’. We mainly focused on articles published within the last decade to ensure the inclusion of the most recent advancements and research findings. To refine the search results, we applied specific inclusion criteria, such as peer-reviewed articles, studies involving human subjects, and papers published in English. Articles that were not directly related to breast cancer imaging or did not involve radiotracers were excluded. Additionally, we reviewed the references of the selected articles to identify any additional relevant studies. This systematic approach ensured a thorough and relevant selection of literature for the review.

## 3. Types of Breast Cancer

### 3.1. Ductal Carcinoma In Situ

Ductal carcinoma in situ (DCIS) is a non-invasive malignancy of the breast characterized by neoplastic proliferation limited to the breast ducts and without myoepithelial invasion. DCIS is a non-obligate precursor to invasive breast cancer, with 25–60% of cases progressing if left untreated [6]. DCIS is one of the most common breast cancers, accounting for 20–25% of all diagnosed breast cancers worldwide [7]. According to estimates by the American Cancer Society, approximately 56,500 new cases of DCIS will be diagnosed in the United States in 2024 [8]. In the majority of cases, with prompt diagnosis and treatment, DCIS has an excellent prognosis and portends a normal life expectancy. In particular, the ten-year overall survival rate is 98% [7], and the twenty-year cancer-specific mortality rate is 3.3% [9].

### 3.2. Invasive Ductal Carcinoma

Invasive ductal carcinoma (IDC) is an invasive malignancy of the breast characterized by the proliferation of neoplastic luminal epithelial cells and their invasion into the surrounding stromal tissue following the disruption of the myoepithelial cell layer, ductal basement membrane, and ductal wall [6]. A significant proportion of patients with IDC have an associated DCIS component, with studies reporting the presence of a DCIS component in 30–60% of cases of IDC [10]. IDC is the most common type of invasive breast cancer, accounting for 70–80% of cases overall [11]. When detected and treated early, IDC has a good prognosis, with a five-year relative survival rate of 90% [11,12].

### 3.3. Invasive Lobular Carcinoma

Invasive lobular carcinoma (ILC) is another invasive malignancy of the breast characterized by a discohesive histopathological phenotype with single-file strands of proliferative neoplastic cells lacking epithelial cadherin (E-cadherin) and infiltrating the stromal and adipose tissues [13]. ILC is the second most common type of invasive breast cancer, accounting for 10–15% of cases worldwide [11]. The five-year relative survival rate in appropriately treated cases is 93% and does not differ significantly from that of IDC [11]. However, the long-term outcomes of ILC are equivalent or even inferior to those of IDC, with the prognosis of ILC becoming progressively worse than that of IDC over time [14].

### 3.4. Triple-Negative Breast Cancer

Breast cancers lacking the expression of estrogen receptor (ER), progesterone receptor (PR), and human epidermal growth factor receptor 2 (HER2) are referred to as triple-negative breast cancers (TNBC) [15,16]. TNBC typically displays an aggressive clinical course, with a poorer prognosis compared to other types of breast cancer, having a five-year relative survival of only 77% after diagnosis compared to 93% in other breast cancer types, as well as a 30–40% five-year recurrence rate after surgical intervention [15,17].

### 3.5. HER2-Positive Breast Cancer

HER2-positive breast cancers are those with overexpression of the HER2 gene with abnormally elevated levels of the HER2 receptor protein [18]. The prognosis of having the HER2 gene is a poor one, with survival rates reducing with each additional copy of the gene [18,19]. Interestingly, HER2 overexpression was found to inversely correlate with hormone receptor (HR) protein expression levels [20]. The five-year survival rate of HER2-positive breast cancer when detected and treated early is 91.5% in cases that are also hormone receptor-positive and 85.7% in cases that are hormone receptor-negative [21]. However, the survival rate decreases to 30% when this aggressive malignancy progresses to more advanced stages [22].

### 3.6. Hormone Receptor-Positive Breast Cancer (Luminal Subtypes)

Luminal A tumors are defined by the presence of ER and/or PR, the absence of HER2, and low levels of the cell proliferation marker Ki-67; in contrast, Luminal B tumors are typically ER-positive and may lack PR, are characterized by high expression of Ki-67, and are of higher grade and have a poorer prognosis compared to Luminal A [23]. Approximately 70–75% of invasive breast cancers exhibit notably elevated ER expression. Meanwhile, PR is found in over 50% of patients who are ER-positive but is extremely rare in those with ER-negative breast cancer [23,24]. The five-year relative survival percentage of women diagnosed with either subtype is relatively high, with that of the luminal A subtype being 94.4%, while those with the luminal B subtype have a slightly lower rate at 90.7% [21].

## 4. Current Radiotracers in Breast Cancer Imaging

### 4.1. ^18^F-Fluorodeoxyglucose (^18^F-FDG)

#### 4.1.1. ^18^F-FDG PET/CT

^18^F-FDG PET/CT imaging plays an important role in diagnosis, staging, prognosis, assessment of recurrence, treatment planning, and response to breast cancer. FDG is a glucose analog transported through a glucose transporter into the cell and phosphorylated by hexokinase. FDG follows a similar pathway to glucose, however, it is not entirely metabolized since it does not have a hydroxyl group at the C-2 position. As a result, it becomes confined in tumor cells at a rate proportional to glucose utilization. The fact that malignant cells have higher glucose metabolism and increased glycolytic activity facilitates their detection using ^18^F-FDG PET/CT imaging [25]. ^18^F-FDG is not very specific for malignancy since its uptake can be seen in inflammatory or infectious conditions. Many benign breast etiologies also show mild increased ^18^F-FDG uptake such as fibroadenoma, ductal adenoma, and fibrocystic changes and these can hence be mistaken for malignancy [26].

##### Ductal Carcinoma In Situ

Tumors like DCIS are often small in size and show low-to-no ^18^F-FDG uptake. In addition, the use of ^18^F-FDG PET/CT to diagnose early-stage breast cancer is limited by its low spatial resolution, which can miss small lesions that are less than 5 mm in size. Therefore, ^18^F-FDG PET/CT is more useful in invasive breast cancer and metastatic disease [26]. Accordingly, the National Comprehensive Cancer Network (NCCN) Clinical Practice Guidelines in Oncology do not recommend the routine use of ^18^F-FDG PET/CT for early diagnosis of breast cancer [27]. However, ^18^F-FDG PET/CT has some utility in cases of DCIS, particularly in predicting the progression of DCIS to invasive cancer. In a study comparing ^18^F-FDG PET/CT imaging findings with histological results from needle biopsy, maximum standardized uptake (SUV_max_, with a cutoff point ≥ 1.9) and tumor size were significant predictors of DCIS upgrade to invasive cancer (*p* = 0.002 and *p* = 0.022, respectively) [28].

##### Invasive Ductal Carcinoma

Studies have shown a higher uptake of ^18^F-FDG in IDC compared to other types of cancer, such as DCIS and ILC, with one study for example showing median SUV_max_ values of 6.6 for IDC and 3.4 for ILC [29,30]. In addition to initial diagnosis, ^18^F-FDG PET/CT plays a crucial role in tumor staging, specifically in invasive breast cancer such as advanced TNBC to assess extra-axillary nodal and distant metastasis. Thus, ^18^F-FDG PET/CT has a significant role in patients at greater risk of metastatic disease [31,32]. Moreover, ^18^F-FDG PET/CT has good performance in detecting bone metastasis secondary to invasive breast cancer. A systematic review and meta-analysis of 668 patients showed that ^18^F-FDG PET/CT performed better than bone scintigraphy with a sensitivity of 93% and a specificity of 99% [33].

^18^F-FDG PET/CT also plays a major role in assessing prognosis, where ^18^F-FDG uptake and SUV_max_ value correlate with tumor invasiveness. A higher SUV_max_ indicates a poorer prognosis and higher disease recurrence [34]. A study by Song et al. concluded that the SUV_max_ value of the primary tumor (pSUV_max_) on pretreatment ^18^F-FDG PET/CT could be used as a good surrogate marker for the prediction of progression in patients with IDC. Meanwhile, patients with a high pSUV_max_ (more than 6.6) had significantly shorter progression-free survival compared to patients with a low pSUV_max_ (*p* < 0.0001) [35].

^18^F-FDG PET/CT can predict treatment response with its ability to identify variations in metabolic activity at earlier stages compared to changes in tumor size detected by morphologic imaging. This is especially helpful in the case of targeted therapy, as treatment can inactivate tumor cells without necessarily affecting their size [25]. Similarly, ^18^F-FDG PET/CT can also predict breast cancer recurrence. A study concluded that ^18^F-FDG PET/CT performed better than contrast-enhanced CT alone or combined with bone scans for evaluating disease recurrence. The results showed an area under the receiver operating curve of 0.99 for ^18^F-FDG PET/CT, 0.84 for contrast-enhanced CT, and 0.86 for combined contrast-enhanced CT and bone scintigraphy [36].

##### Invasive Lobular Carcinoma

Accurate staging of ILC, a subtype of breast cancer, is crucial for successful treatment. While ^18^F-FDG PET/CT is commonly used for this purpose, its effectiveness varies depending on the histologic subtype. For instance, identifying ILC using ^18^F-FDG PET/CT is challenging due to its distinctive molecular and clinical characteristics, such as decreased cellular density, receptor expression, and different metastatic patterns. Additionally, the absence of E-cadherin leads to dispersed tumor cells and a low rate of tumor-infiltrating lymphocytes [37]. These unique attributes of ILC diminish the sensitivity of ^18^F-FDG PET/CT in detecting cancer due to the dispersion of glucose-intensive tumor tissues. Consequently, both primary and metastatic ILC have lower ^18^F-FDG levels than IDC, limiting the efficacy of ^18^F-FDG PET/CT for detecting ILC [38]. Moreover, research has shown that in newly diagnosed stage I-III ILC patients, ^18^F-FDG PET/CT has a lower impact on systemic staging compared to IDC patients. Additionally, untreated bone metastases in IDC patients tend to have higher ^18^F-FDG uptake, while non-FDG-avid sclerotic bone metastases are more common in ILC. Therefore, it is recommended to carefully evaluate CT images from PET/CT scans for ILC patients [39].

When it comes to detecting recurrence, ^18^F-FDG PET/CT demonstrated usefulness in the surveillance of ILC. The sensitivity, specificity, positive predictive value (PPV), and negative predictive value (NPV) of PET/CT for predicting recurrence in ILC were 87%, 87%, 95%, and 70%, respectively [40]. ILC recurrence sites had high SUV_max_ values, with a mean SUV_max_ of 6.4. In 40 patients with histopathological data, 30 PET/CT scans yielded true positive results. However, false negatives were reported in cases of local, peritoneal, meningeal, and bladder recurrences [40]. Moreover, in the first instance of ILC recurrence, the detection of an axillary lymph node using ^18^F-FDG PET/CT even when ^18^F-FDG uptake is low, showed an approximate sensitivity of 52%, specificity of 87%, and accuracy of 85%. ^18^F-FDG PET/CT demonstrated significantly higher specificity compared to MRI with 88% specificity [41]. Overall, ^18^F-FDG PET/CT findings influenced treatment plans for 92% of patients [40].

##### Triple-Negative Breast Cancer

Advances in ^18^F-FDG PET/CT have played a revolutionary role in diagnosing, staging, and treating TNBC, a highly aggressive form of breast cancer and thus metastasizes very early on. The use of ^18^F-FDG PET/CT in tumor staging in breast cancer, notably TNBC, is essential. Effective detection of distant metastases is crucial for patient management as it changes management from surgery with or without neoadjuvant systemic therapy to palliative systemic therapy without surgery [42].

^18^F-FDG PET/CT provides better predictive stratification than traditional imaging as metabolic uptake varies with tumor phenotype. For instance, in TNBC, high ^18^F-FDG uptake indicates increased metabolic activity and may predict treatment outcomes. A multivariate regression analysis of TNBC cases showed significant positive correlations between SUV_max_ and tumor size (*p* = 0.009) as well as between SUV_max_ and Ki-67 score (*p*  <  0.001), indicating a wide range of glucose metabolism. This demonstrates that ^18^F-FDG PET/CT imaging could be used to measure the Ki-67 proliferation index and identify aggressive TNBC cases [43].

**Figure 1:**
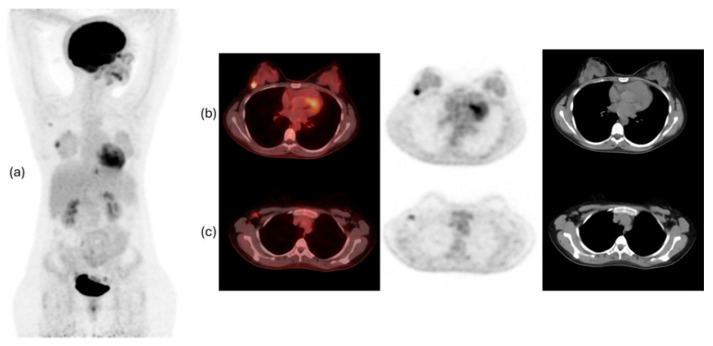
^18^F-fluorodeoxyglucose (^18^F-FDG) positron emission tomography/computed tomography (PET/CT) images of a 37-year-old woman with invasive ductal carcinoma of the right breast, showing an FDG-avid lesion in the right breast in keeping with the primary malignancy (**a**,**b**), as well as an FDG-avid right axillary lymph node, which is likely metastatic (**a**,**c**).

**Figure 2:**
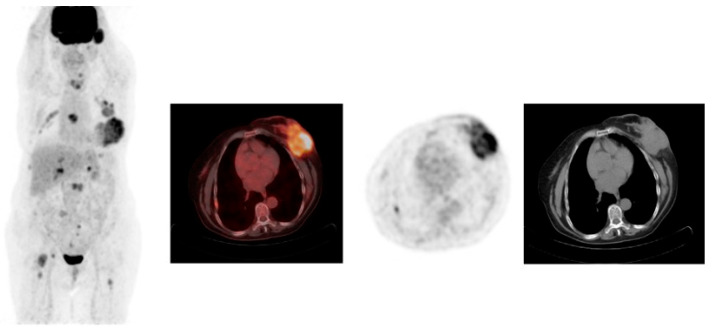
^18^F-FDG PET/CT images of a 77-year-old woman with metastatic left breast cancer. The images show a large FDG-avid lobulated necrotic mass in the outer aspect of the left breast, corresponding to the primary malignancy. There are enlarged FDG-avid metastatic left axillary and retropectoral lymph nodes, FDG-avid metastatic subcutaneous soft tissue deposits, and multiple innumerable intensely FDG-avid metastatic lytic bone lesions.

Figure 1 and Figure 2 show examples of ^18^F-FDG PET/CT imaging of breast cancer, with the former showing a case of locoregional malignancy and the latter showing a case of metastatic malignancy.

#### 4.1.2. ^18^F-FDG Positron Emission Mammography (PEM)

As mentioned previously, ^18^F-FDG PET/CT can be used in clinical settings for staging, re-staging, and assessment of therapeutic response in cases of breast cancer; however, because of its limited spatial resolution especially for sub-centimetric lesions, this technique is not recommended as a means of detecting primary breast cancer or discriminating between benign and malignant breast lesions. These limitations led to the development of different breast imaging devices and nuclear imaging techniques such as positron emission mammography (PEM) to address the shortcomings of ^18^F-FDG PET/CT. Unlike the whole-body imaging performed in traditional PET/CT scans, PEM is a breast-specific positron imaging system that employs a device which brings the detectors closer to the breast in order to achieve dedicated high-resolution images, with the breast immobilized under gentle compression as the image is being taken. The most common radiotracer employed in PEM is also ^18^F-FDG. Given that PEM involves imaging the breasts, its utility is mainly in the diagnosis and assessment of primary malignancies rather than distant metastases, which are better evaluated using whole-body PET/CT [44].

In a study comparing PEM with the standard of X-ray mammography, the sensitivity, specificity, accuracy, PPV, and NPV of PEM in diagnosing various types of breast cancer were 90%, 86%, 88%, 88%, and 88%, respectively (compared with the respective values of 92%, 48%, 71%, 57%, and 84% with conventional mammography). In addition, studies have shown that PEM is superior to mammography in several aspects such as distinguishing between benign and malignant lesions, not requiring the use of X-ray radiation, yielding a more rapid diagnosis, offering high-precision information about the location of lesions, and identifying suspicious lesions in the breast [45,46]. A preliminary study on breast cancer screening using PEM showed a cancer detection rate of 2.3%, compared with a cancer detection rate of approximately 0.2% using whole-body PET imaging and 0.31% using mammography and physical examination. This result meets the international screening guidelines and shows that PEM may have potential as a screening tool in conjunction with X-ray mammography [47].

In view of the fact that PEM has a better spatial resolution and takes less time than PET/CT, it may be a useful diagnostic tool and has been investigated for this purpose in comparison with other imaging modalities [45]. In regard to diagnosis, PEM has shown equivalent sensitivity to MRI with potentially higher specificity for detecting abnormalities. In a pilot study of 25 patients with newly diagnosed breast cancer, PEM detected 96% of malignant index lesions. Despite the small sample size, PEM resulted in fewer false-positive extra lesions than MRI [48]. Similarly, for preoperative local staging, two prospective studies have shown equivalent sensitivity and superior specificity of PEM in comparison with MRI, with one study reporting that PEM had a sensitivity of 80.5% and specificity of 91.2% compared with the respective values of 80.7% and 86.3% using MRI [49,50]. In comparison with PET/CT, PEM has demonstrated superior results in the diagnosis of breast cancer, with a meta-analysis showing higher sensitivity, accuracy, and NPV of PEM, and equivalent specificity and PPV between the two modalities [45]. PEM has also proven to be more effective than whole-body PET/CT in the detection of residual disease after the completion of NAC. For example, one study using PEM to predict response to NAC showed a sensitivity, specificity, and accuracy of 77.1%, 83.3%, and 78.7%, respectively, compared with the respective values of 54.3%, 83.3%, and 61.7% using whole-body PET imaging [51,52].

Overall, PEM is a breast-specific molecular imaging modality that has great potential as a tool for specific uses such as local staging, evaluation of the response to neoadjuvant treatment, and detection of local disease recurrence [44], in addition to its utility as a diagnostic tool and perhaps even as a screening tool. PEM is also advantageous in that it can be used to biopsy specific lesions, and a PEM-guided biopsy system is available in the market that employs a stereotactic method for targeting lesions [53]. However, the radiation exposure involved in PEM must be taken into consideration. In comparison with X-ray mammography, PEM is associated with a 15-fold increase in the risk of cancer induction and a 25-fold increase in the overall risk of cancer-related mortality. This relates to the fact that only the fibroglandular breast tissue is exposed to significant amounts of ionizing radiation in X-ray mammography, resulting in a risk of inducing breast cancer, whereas PEM involves the use of radionuclides which can induce malignancy in any radiosensitive organs. In particular, the bladder has the highest radiation dose and risk of cancer induction in PEM [54].

**Figure 3:**
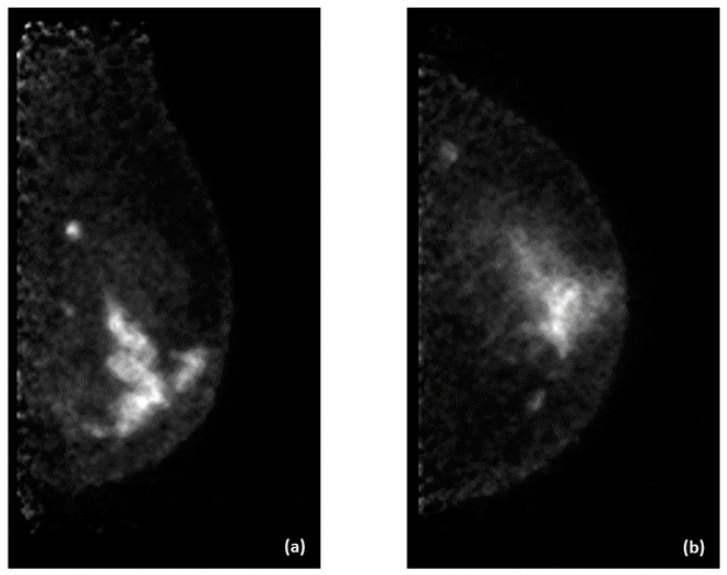
3′-deoxy-3′-[^18^F]-fluorothymidine (^18^F-FLT) positron emission mammography (PEM) images of a 47-year-old woman with left breast cancer. (**a**) Left mediolateral oblique (LMLO) view. (**b**) Left craniocaudal (CC) view.

**Figure 4:**
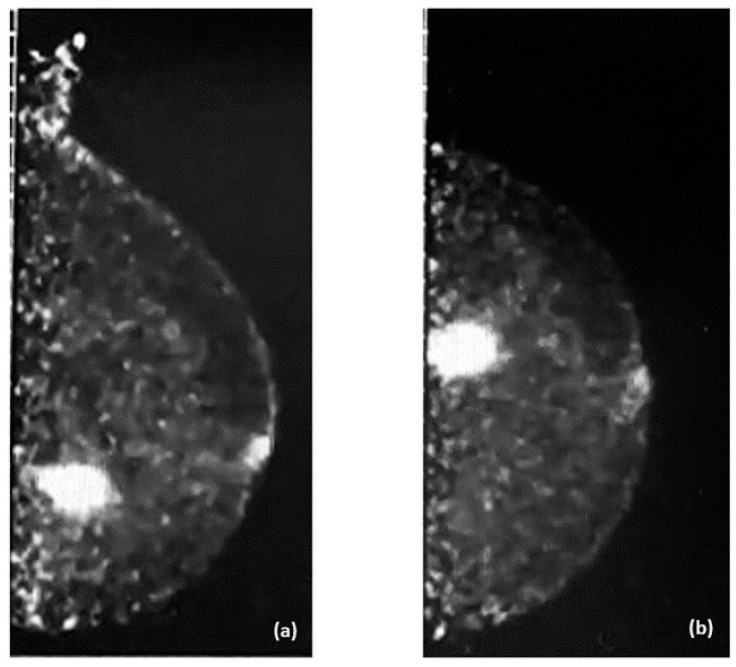
^68^Ga-Trivehexin positron emission mammography (PEM) images of a 57-year-old woman with left breast cancer. (**a**) Left mediolateral oblique (LMLO) view. (**b**) Left craniocaudal (CC) view.

Examples of PEM images of breast cancer are shown in Figure 3 and Figure 4, with the former showing PEM using 3′-deoxy-3′-[^18^F]-fluorothymidine (^18^F-FLT) and the latter showing PEM using ^68^Ga-Trivehexin.

#### 4.1.3. ^18^F-FDG PET/MRI

There is great potential for integrating PET with MRI. In a prospective study by Moy et al. with 36 women with confirmed or suspected breast cancer, researchers found that while breast MRI alone was highly sensitive (96%), combining it with prone-position [^18^F]FDG-PET/CT data increased the PPV from 77% to 98% and the specificity from 53% to 97%. The overall accuracy also improved from 78% with MRI alone to 89% with the fused PET/MRI [55]. In a study by Botsikas et al., in the lesion-per-lesion analysis, the sensitivity of PET/MR compared to PET/CT for detecting bone metastases, other metastases, axillary and internal mammary nodes, and contralateral tumors combined was 89% vs. 77% (*p* = 0.0013); Meanwhile, the corresponding specificity was 96% vs. 98% (*p* = 0.0075) [56]. A meta-analysis done by Zhang et al. has shown that PET/MRI has higher accuracy in the distant staging of breast cancer compared to PET/CT, with a sensitivity of 95% vs. 87%, all the while having comparable specificities of 96% vs. 94% [57].

PET/MRI has been investigated for its role in diagnosing, staging, and phenotyping breast cancer, as well as assessing prognosis and treatment response. It offers a comprehensive evaluation for newly diagnosed patients by identifying disease spread throughout the body; it can also provide critical information that influences clinical management and exposes patients to significantly less radiation than PET/CT. However, the studies are limited by their sample sizes. For now, PET/MRI is most suitable for patients who require both PET/CT and MRI for disease evaluation, as PET/MRI offers a two-in-one solution [56,58].

### 4.2. Fibroblast Activation Protein Inhibitor (FAPI)

Another important radiotracer is the fibroblast activation protein inhibitor (FAPI). The fibroblast activation protein (FAP) is a type II transmembrane serine protease. Its expression is increased in cancer-associated fibroblasts (CAFs), which induces tumor proliferation and worsens the survival of cancer patients. Cancer-associated fibroblasts have FAP as a target, which is highly specific to tumor expression. ^68^Ga-FAPI and ^18^F-FAPI are among the many FAP-targeting radiotracers developed that play a significant role in tumor detection. However, ^68^Ga has a short half-life of 68 min and a lower image resolution compared to ^18^F which has a longer half-life of 110 min as well as a higher image resolution [25,59].

When comparing ^68^Ga-FAPI to ^18^F-FDG in diagnosing breast cancer, studies have shown that ^68^Ga-FAPI had a higher overall tumor uptake in breast cancer and a higher metastatic detection rate than ^18^F-FDG specifically when metastasis was in the bone and peritoneum [60,61,62,63]. One of the disadvantages of ^18^F-FDG is its low specificity since benign conditions such as infection, fibroadenoma, ductal adenoma, inflammatory granulomatous mastitis, and fibrocystic changes are also FDG-avid [33]. In such cases, using ^68^Ga-FAPI might be a better choice in breast cancer staging since it is more specific and has a decreased uptake in the brain, liver, and oral mucosa [64].

**Figure 5:**
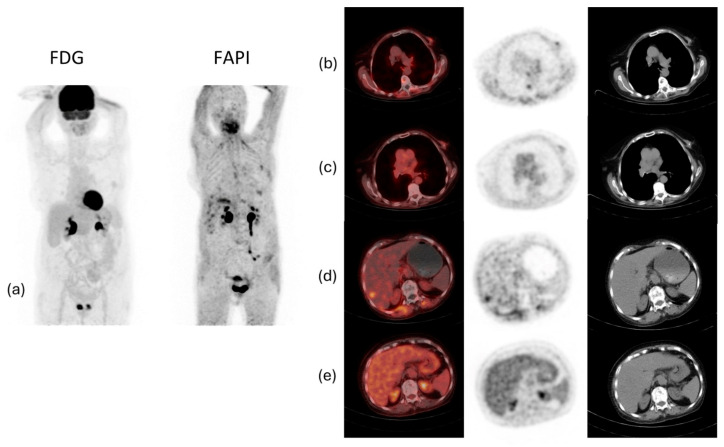
^18^F-FDG and ^68^Ga-fibroblast activation protein inhibitor-46 (^68^Ga-FAPI-46) PET/CT images of a 74-year-old woman with invasive lobular carcinoma of the left breast. (**a**) Maximum intensity projection (MIP) whole-body PET/CT images. (**b**) ^68^Ga-FAPI-46 PET/CT of the primary breast lesion. (**c**) ^18^F-FDG PET/CT of the primary breast lesion. (**d**) ^68^Ga-FAPI-46 PET/CT of the metastatic hepatic lesions. (**e**) ^18^F-FDG PET/CT of the metastatic hepatic lesions. The images show faint ^18^F-FDG uptake in the left breast, increased ^68^Ga-FAPI uptake in the upper central quadrant of the left breast, ^68^Ga-FAPI-46 avid metastatic hepatic lesions, and ^68^Ga-FAPI-46 avid pleural thickening in the right lower lobe of the lung (likely atelectatic).

Studies have also demonstrated that ^68^Ga-FAPI PET/CT is superior to ^18^F-FDG PET/CT in ILC by showing higher tumoral activity and tumor-to-background uptake ratios, and by detecting more primary tumors, axillary lymph node metastases, and additional foci, including multicentric cancer. Furthermore, ^68^Ga-FAPI PET/CT detected more bone and liver metastases, and a positive association was made between the peritumoral lymphocyte ratio and ^68^Ga-FAPI PET/CT-to-^18^F-FDG PET/CT uptake ratios [38]. ^68^Ga-FAPI PET/CT showed increased effectiveness in detecting lesions in the breast, lymph nodes, lung, liver, and bone [65,66]. Moreover, compared to CT alone, ^68^Ga-FAPI PET/CT detected more lesions, especially in infiltrative soft tissue and serosal locations [65]. However, larger cohorts are needed to assess these findings further. Figure 5 shows an example of a case of metastatic ILC with a comparison between ^18^F-FDG PET/CT and ^68^Ga-FAPI PET/CT, demonstrating that the latter was better able to detect the primary malignancy as well as the metastatic lesions.

### 4.3. 16α-[^18^F]fluoroestradiol (FES)

Estrogen receptors are elevated in 70–75% of breast cancers, making estrogen receptor radiotracers highly valuable in both disease prognosis and prediction [23,24]. 16α-[^18^F]fluoroestradiol (FES) is a radiotracer for estrogen receptors in breast cancer and is regarded as the first PET imaging agent used for a receptor target in cancer, discovered in 1984 and Food and Drug Administration (FDA)-approved in May 2020 [67,68].

Since most breast cancers are estrogen receptor-positive (ER+), making hormonal therapy important, there is only a 30–40% response rate in metastatic cases. PET imaging using FES can identify candidates for antiestrogen therapy by assessing receptor function. Increased tumor uptake of FES predicts responsiveness to antiestrogen therapy, while a lack of FES uptake indicates potential hormone resistance. A study by Martimer et al. aimed to determine if ^18^F-FDG and FES can detect hormone-induced changes in tumor metabolism and ER levels before and after tamoxifen treatment, and if these PET findings could predict hormonally responsive breast cancer. Forty women with advanced ER-positive breast cancer underwent PET scans with ^18^F-FDG and FES before and 7 to 10 days after starting tamoxifen therapy, evaluating seventy lesions using the mean standardized uptake value (SUV_mean_). Responders showed a 28.4% increase in tumor ^18^F-FDG uptake after tamoxifen, with only five exhibiting a clinical flare reaction, while non-responders had no significant change in tumor ^18^F-FDG uptake. Responders had higher baseline FES uptake (SUV_mean_ 4.3) compared to non-responders (SUV_mean_ 1.8). All patients showed ER blockade after tamoxifen initiation, with a greater degree in responders (54.8% decrease) than in non-responders (19.4% decrease) [69].

This concludes that a decrease in FES uptake after tamoxifen treatment suggests effective ER binding and is in agreement with other similar studies [69,70,71,72]. The baseline SUV_mean_ for FES and changes post-therapy can help identify hormone responsiveness in breast cancer [69,70]. With an SUV_mean_ cutoff of 2.0 for baseline FES-PET, the PPV is 79% and the NPV is 88%; this suggests that FES-PET offers much greater predictive values for hormone therapy responsiveness compared to standard clinical measurement of ER by IHC, which has a predictive value of only 50% in ER-positive patients [67].

FES-PET imaging can also provide prognostic information. Kurland et al. evaluated progression-free survival in 84 patients with ER-positive/HER2-negative breast cancer receiving endocrine therapy, using lesion uptake from ^18^F-FDG and FES-PET. Patients were divided into three groups: low ^18^F-FDG uptake (29%), FDG-avid tumors with high FES uptake (59%), and FDG-avid tumors with low FES uptake (12%). The median progression-free survival for these groups was 26.1 months, 7.9 months, and 3.3 months, respectively. The study indicated that ^18^F-FDG PET and FES-PET might provide important prognostic information, helping identify patients with indolent disease and aiding in the selection of targeted and/or cytotoxic chemotherapy [73].

Moreover, metastatic breast tumors may become heterogeneous, affecting responsiveness to treatment [74]. In a study conducted by Gennari et al., eligible patients underwent an ^18^F-FES PET/CT at baseline, with those having SUV_mean_ ≥ 2 receiving single-agent endocrine therapy until disease progression, and those with SUV_mean_ < 2 being randomized to either endocrine therapy or chemotherapy to compare the activity of first-line endocrine therapy versus chemotherapy in patients with SUV_mean_ < 2. One hundred and seventeen received ET and 30 were randomized to endocrine therapy or chemotherapy. After a median follow-up of 62.4 months, 73.2% experienced disease progression and 37.3% died. Median progression-free survival was 12.4 months for patients with SUV_mean_ < 2 who took endocrine therapy and 23.0 months for those who took chemotherapy, while median progression-free survival was 18.0 months for patients with SUV_mean_ ≥ 2 on endocrine therapy. The ET-FES trial showed that ER+/HER2- metastatic breast cancer patients have varying levels of endocrine responsiveness based on ^18^F-FES PET/CT SUV_mean_ [75]. When evaluating the impact of FES-PET on staging, a study done by Gupta et al. on 12 patients with 154 disease lesions found that ^18^F-FES PET-CT provided better characterization of lesions and influenced management decisions in 20% of patients [76].

**Figure 6:**
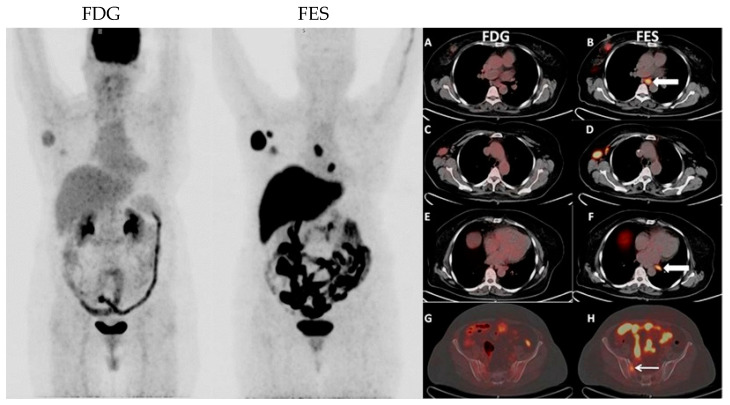
Well-differentiated estrogen receptor (ER)-positive breast tumor with significantly less radiotracer uptake on ^18^F-FDG PET/CT than on 16α-[^18^F]fluoroestradiol (FES) PET/CT, with FES showing more sites of ER-positive disease. The images show FES-avid mediastinal lymph nodes and right iliac bone lesion (**B**,**D**,**F**,**H**; white arrows in FES images) with significantly less ^18^F-FDG uptake (**A**,**C**,**E**,**G**).

**Figure 7:**
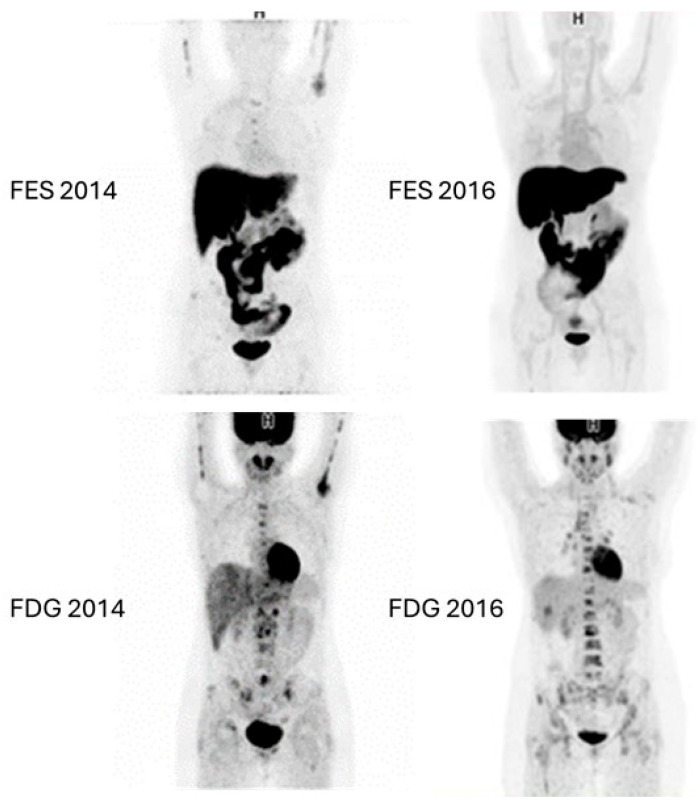
^18^F-FDG and FES PET/CT images of a 36-year-old woman diagnosed with breast cancer. The images from 2014 show matched lesions seen with both ^18^F-FDG and FES. The patient subsequently received multiple lines of hormone-targeted therapy. The images from 2016 after treatment show that FES was negative, but ^18^F-FDG avid metastatic lesions were detected in the lymph nodes, bones, and liver. The images show the gradual transformation from functional ER-positive to ER-negative tumors. These serial images show the paradigm transformation and hence an impact on management and treatment.

Figure 6 and Figure 7 show examples of images of breast cancer obtained using FES PET/CT.

### 4.4. 4-Fluoro-11β-Methoxy-16α-^18^F-Fluoroestradiol (^18^F-4FMFES)

As previously shown, ^18^F-FES is extremely useful for whole-body monitoring of estrogen receptor status in both breast and gynecologic cancers; however, it does come with some shortcomings. ^18^F-FES is quickly metabolized in the liver, creating low-affinity radio metabolites that increase nonspecific background activity, especially in the mediastinal region, reducing image quality. ^18^F-FES is a steroid-based tracer; hence, it has high hepatic uptake and biliary excretion. This leads to high background activity in the liver and intestines, making lesion detection difficult. Additionally, ^18^F-FES binds to plasma globulins, such as sex hormone-binding globulin (SHBG) and albumin. The level of SHBG inversely affects tumoral ^18^F-FES uptake, as globulin-bound ^18^F-FES cannot target receptors, contributing to the blood pool [77].

To improve ^18^F-FES, researchers modified the parent estradiol molecule. Several 11b-methoxy or A-ring fluorine-substituted ^18^F-FES derivatives were synthesized. Among these, 4-fluoro-11β-methoxy-16α-^18^F-FES (^18^F-4FMFES) showed the highest uterine and brain uptake as well as best uterus-to-blood ratios in female rats as well as a lower affinity for plasma globulins [78,79,80]. Further studies in tumor-bearing mice revealed that ^18^F-4FMFES had the best in vivo ER1 tumor uptake and image contrast [78]. A phase I clinical study in healthy women showed substantial uterine uptake and retention, faster blood clearance, and lower nonspecific organ uptake compared to ^18^F-FES [79].

Paquette et al. conducted a phase II study on newly diagnosed ER1 breast cancer patients to compare the performance of ^18^F-4FMFES PET with ^18^F-FES PET. The study included time-dependent blood metabolite analysis and static PET imaging using both tracers within a 7-day interval. The study demonstrated that ^18^F-4FMFES PET provides a lower nonspecific signal and better tumor contrast compared to ^18^F-4FMFES PET, resulting in improved diagnostic confidence and fewer false-negative diagnoses [81].

### 4.5. 21-^18^F-Fluoro-16α,17α-[(R)-(1′-α-Furylmethylidene)dioxy]-19-Norpregn-4-ene-3,20-Dione (FFNP)

21-^18^F-fluoro-16α,17α-[(R)-(1′-α-furylmethylidene)dioxy]-19-norpregn-4-ene-3,20-dione, also known simply as ^18^F-FFNP, is a radiotracer with high affinity and selectivity for the PR [82]. Out of several progestin derivatives, ^18^F-FFNP was shown to be the most successful for PET imaging based on preclinical studies, designed to be stable against defluorination, resulting in low bone uptake studies. Importantly, due to its relatively low lipophilicity, it accumulates minimally in the liver and fat, leading to low nonspecific binding in vivo [83,84,85,86]. As mentioned earlier, decreases in FES uptake after starting ER antagonist therapy, such as tamoxifen or fulvestrant, confirm that the drug effectively targets the receptor, as the antagonist occupies the receptor-ligand binding pocket over FES. However, antagonist binding does not ensure complete inhibition of ER transcriptional function, especially with ESR1 gene mutations [87]. This has led to exploring imaging of estrogen-regulated target genes, like PR, to monitor therapy response. In a study conducted by Dehdashti et al., ^18^F-FFNP PET was shown to be effective in assessing the PR status of individual breast cancer lesions, with the potential to noninvasively and repeatedly evaluate the PR positivity of lesions, aiding in the decision-making for antiestrogen therapy before or after endocrine treatment [88]. In another study by Dehdashti et al., they demonstrated that the estradiol-challenge test using FFNP-PET effectively predicts response to endocrine therapy (ET) in advanced ER+ breast cancer patients, with a >6.7% increase in FFNP uptake accurately identifying responders, with 100% PPV and NPV. This method outperforms ^18^F-FDG PET in heavily pretreated subjects and offers a rapid, reliable prediction of ET benefit within 2 days, showing significant separation between responders and non-responders. Additionally, it correlates with longer overall survival in responders and efficiently identifies non-responders in patients receiving CDK4/6 inhibitor/ET therapy [89].

### 4.6. ^89^Zr-Trastuzumab

Numerous radiotracers targeting HER2-positive malignancies have been reported in the literature, with the strongest evidence supporting the use of ^89^Zr-trastuzumab. Zirconium-89 is a positron-emitting radiometal with a long and favorable half-life of 78.4 h, making it compatible with the half-life of trastuzumab in vivo and hence a suitable radionuclide for use in this setting. ^89^Zr-labeled antibodies can be visualized for up to seven days after injection; however, optimal PET scanning should be done 4–5 days after injection. ^89^Zr-trastuzumab is subject to high uptake in regions of high perfusion and vascularity, thereby making it particularly suited for the imaging of metastatic lesions characterized by vigorous angiogenesis. In one study, HER2-positive metastatic lesions demonstrated significant radiotracer uptake, and previously unknown brain metastases were detected [90]. In other studies, HER2-positive metastatic lesions were detected in patients with HER2-negative primary malignancies and vice versa, highlighting the potential utility of ^89^Zr-trastuzumab as a tool to investigate the heterogeneity between primary and metastatic lesions [42,91]. Another study showed that ^89^Zr-trastuzumab PET/CT had a PPV of 88% and an NPV of 72% in the prediction of morphological response of metastatic breast cancer to treatment with trastuzumab emtansine, compared with 83% and 96%, respectively, for ^18^F-FDG PET/CT. The combination of ^89^Zr-trastuzumab PET/CT and ^18^F-FDG PET/CT yielded a PPV and NPV of 100%, accurately predicting morphological response to treatment [92].

### 4.7. ^64^Cu-DOTA-Trastuzumab

A similar radiotracer is ^64^Cu-DOTA-trastuzumab, which has a half-life of 12.7 h and can therefore achieve high-resolution imaging with lower radiation exposure and in a shorter time frame. An early study in 2013 showed that ^64^Cu-DOTA-trastuzumab PET/CT imaging was successful in detecting HER2-positive primary malignant lesions. In addition, brain metastases were detected in some patients, indicating adequate infiltration via the blood–brain barrier [93]. Another study showed that ^64^Cu-DOTA-trastuzumab PET/CT had a sensitivity of 89% when imaging was done on day 2 post-injection, which was comparable to the sensitivity of 93% achieved using traditional ^18^F-FDG PET imaging. This study showed that the ideal imaging time should be 48 h after the injection of the radiotracer and that ^64^Cu-DOTA-trastuzumab is highly sensitive for the detection of HER2-positive malignancies and the surveillance of disseminated disease. Furthermore, some patients were pre-treated with trastuzumab prior to imaging, which improved the biodistribution of the radiotracer and the imaging quality by reducing liver uptake by 75% without affecting tumor uptake [94]. A third study further confirmed the effectiveness of ^64^Cu-DOTA-trastuzumab PET/CT imaging in detecting primary lesions with a strong correlation with histopathological HER2 status, and also showed notable uptake in metastatic liver lesions and was able to detect these metastases. However, in this study, significant variability in SUV_max_ was detected within and among HER2-positive patients, and an overlap was seen between HER2-positive and HER2-negative patients [95]. Interestingly, a recent study investigated the use of ^64^Cu-DOTA-trastuzumab PET imaging to predict disease response to treatment with trastuzumab emtansine. Patients with HER2-positive metastatic disease were enrolled and underwent ^64^Cu-DOTA-trastuzumab PET imaging on days 1 and 2 preceding treatment. Favorable response to treatment was correlated with specific SUV_max_ values, specifically the day 2 minimum SUV_max_ value [96].

### 4.8. ^89^Zr-Pertuzumab

Alongside trastuzumab, pertuzumab is another monoclonal antibody often used for the treatment of metastatic HER2-positive breast malignancies. Accordingly, a radiolabeled tracer employing pertuzumab tagged with zirconium-89 has been developed and is under study in the literature. The first human study in 2018 showed that the optimal time for imaging is 5–8 days after administration of ^89^Zr-pertuzumab, which allowed sufficient time for the elevated background radioactivity in the liver and blood to decrease. In all patients with HER2-positive primary malignancies, the primary lesions showed avid uptake of ^89^Zr-pertuzumab. In addition, metastatic brain lesions showed avid uptake of ^89^Zr-pertuzumab. Similarly, ^89^Zr-pertuzumab PET/CT imaging detected a HER2-positive metastatic lesion in the chest. However, other metastatic lesions were not detected; for instance, lung and nodal metastases only showed mild avidity, and liver metastases did not show significant avidity. Interestingly, ^89^Zr-pertuzumab also showed utility in the investigation of heterogeneity in HER2 status and was able to distinguish between HER2-positive and HER2-negative lesions within the same patient [97]. Another study further investigated the use of ^89^Zr-pertuzumab in patients with heterogeneous lesions and showed that it was able to detect some HER2-positive metastatic lesions in patients with HER2-negative primary malignancies [98]. A site specifically-labeled conjugate known as ^89^Zr-site-specific (ss)-pertuzumab has also been investigated. A recent study showed that ^89^Zr-ss-pertuzumab yields improved lesion detection owing to increased tracer avidity, but again failed to detect significant metastases in the liver, bones, lungs, and other sites; its utility in detecting brain metastases is yet to be studied. The study also showed that radiotracer imaging can directly impact therapeutic considerations; one patient with HER2-positive primary malignancy and diffuse osseous and hepatic metastases demonstrated minimal foci of avid ^89^Zr-pertuzumab uptake and was thus switched to chemotherapy with doxorubicin instead of HER2-targeted therapy [99].

## 5. Investigational Radiotracers in Breast Cancer Imaging

### 5.1. [^18^F]PSMA-1007

Patients with TNBC have limited treatment options because their tumors do not have human epidermal growth factor receptors, progesterone receptors, or estrogen receptors. Thus, understanding the TNBC microenvironment and identifying molecular subgroups can provide immunotherapy benefits. Recent studies have shown increased levels of prostate-specific membrane antigen (PSMA) in tumor-associated neovascularization and metastases, indicating the potential for both therapy and diagnosis [100]. For instance, a prospective cohort study limited by the small number of patients demonstrated that TNBC lesions can be successfully detected using [^18^F]PSMA-1007 PET/CT [101]. Moreover, compared to standard ^18^F-FDG PET/CT, [^18^F]PSMA-1007 demonstrated higher accumulation in distant metastases, especially in the brain, potentially providing benefits in detecting distant metastases. In addition to that, the high presence of PSMA in TNBC, as revealed by immunohistochemical investigations, suggests that it could be a target for treatment. PSMA-based imaging is promising, especially for TNBC patients, despite ^18^F-FDG PET/CT still being more sensitive [100]. Further research is necessary to fully assess these results, as PSMA-based radiopharmaceuticals may enhance TNBC diagnostic imaging and potentially open up new treatment possibilities.

### 5.2. ^68^Ga-ABY-025

A recently emerging radiotracer is ^68^Ga-ABY-025, which consists of the biopharmaceutical HER2-binding Affibody molecule ABY-025 tagged with gallium-68. A study showed that ^68^Ga-ABY-025 PET/CT imaging was able to detect HER2-positive primary malignancies effectively. In regard to metastatic lesions, ^68^Ga-ABY-025 imaging was most effective in detecting liver metastases which showed more avid uptake than any other metastatic lesions [102]. A subsequent study investigated the use of ^68^Ga-ABY-025 imaging to predict metabolic response to treatment. The results showed a significant negative correlation between ^68^Ga-ABY-025 SUV_max_ and the change in tumor lesion glycolysis (Δ-TLG). This shows that ^68^Ga-ABY-025 imaging may be useful as an adjunct tool to estimate the level of HER2 expression needed to induce metabolic remission using HER2-targeted therapies [103]. In a recent study in 2024, some patients had avid uptake in 2–5 mm lymph nodes that had not shown radioactivity on ^18^F-FDG PET/CT scans, which led to re-staging and a change in the treatment regimen. This indicates the potential utility of ^68^Ga-ABY-025 imaging in accurate disease staging. In addition, ^68^Ga-ABY-025 imaging reduced false positive results; patients who had recently received the COVID-19 vaccine had avid uptake of ^18^F-FDG in lymph nodes ipsilateral to the vaccination site, whereas no uptake of ^68^Ga-ABY-025 was seen in these benign inflammatory lesions [104].

### 5.3. ^68^Ga-NOTA-Fab-Trastuzumab

A study in 2022 showed that the molecule ^68^Ga-NOTA-Fab-trastuzumab had a favorable pharmacokinetic profile, with decreased non-specific binding and relatively low uptake in the liver and non-tumoral regions. Notably, ^68^Ga-NOTA-Fab-trastuzumab PET/CT imaging showed avid uptake in HER2-positive primary lesions as well as in metastatic lesions in the axillary lymph nodes. However, because of the elevated physiological uptake in the blood pool and liver, metastatic lesions in the mediastinal lymph nodes near the heart as well as in the liver could not be detected [105,106]. A recent study in 2023 investigated the utility of ^68^Ga-DFO-Fab-trastuzumab-M74, a similar radiotracer using DFO instead of NOTA as a chelating agent and with the addition of a methionine residue M74. In vitro cell studies and in vivo mouse studies showed that the M74-conjugated radiotracer had a better pharmacokinetic profile with higher affinity to HER2-expressing cells, resulting in more rapid blood clearance, less liver uptake, and more tumor uptake [107].

### 5.4. ^89^Zr∙Df-HER2-Fab-PAS200

An emerging radiotracer that has been preliminarily tested is ^89^Zr∙Df-HER2-Fab-PAS200. This molecule consists of a PASylated antibody fragment tagged with zirconium-89. In particular, PASylation is a process of genetic fusion that produces functional proteins with attached sequences of proline (P), alanine (A), and serine (S) amino acids; this technique increases the size of the fusion protein and hinders its clearance, thereby resulting in a prolonged half-life and enhanced, longer-lasting activity. In the first human study, a 67-year-old woman with HER2-positive breast cancer metastatic to the axillary lymph nodes and brain underwent ^89^Zr∙Df-HER2-Fab-PAS200 PET/CT imaging. The radiotracer showed strong uptake and signal in the primary malignant lesion and the sites of lymph node metastasis. However, in contrast with other radionuclides such as ^89^Zr-trastuzumab, no radioactivity was seen in the metastatic brain lesions, possibly because the patient had been pre-treated with dexamethasone which may have stabilized the blood–brain barrier and prevented the penetration of ^89^Zr∙Df-HER2-Fab-PAS200 [108].

### 5.5. ^68^Ga-HER2-Nanobody

An additional radiotracer that is currently being studied is the ^68^Ga-HER2-Nanobody, also known as ^68^Ga-NOTA-anti-HER2-sdAb. Nanobodies are antigen-specific single-domain antibodies (sdAbs) that consist of a monomeric variable domain derived from heavy-chain antibodies. An initial study in 2016 showed that ^68^Ga-HER2-Nanobody is characterized by accelerated blood clearance, which is advantageous and allows for rapid same-day imaging of patients. Additionally, the results showed avid uptake in most metastatic lesions, including those in axillary, hilar, and mediastinal lymph nodes and the pelvic region among other sites. However, the primary lesions showed a heterogeneous uptake pattern, indicating that this radiotracer may be more suited for the assessment of metastatic lesions and not ideal for detecting HER2 expression in primary breast lesions [109]. In a follow-up study in 2024, ^68^Ga-NOTA-anti-HER2-sdAb PET imaging was better able to detect inter-lesional heterogeneity than ^18^F-FDG PET imaging and showed uptake in some malignant lesions with low proliferation and metabolic activity which had not shown ^18^F-FDG uptake. In addition, avid uptake of ^68^Ga-NOTA-anti-HER2-sdAb in the lymph nodes and bones of several patients revealed additional metastases that had not been detected using ^18^F-FDG [110].

### 5.6. ^68^Ga-NOTA-MAL-Cys-MZHER_2:342_

Another emerging radiotracer for the detection of HER2-positive malignancy is ^68^Ga-NOTA-MAL-Cys-MZHER_2:342_, which consists of the HER2-targeting antibody ZHER_2:342_ conjugated with NOTA-MAL and radiolabeled with gallium-68. An initial study showed avid in vitro uptake of the radiotracer in HER2-positive cell lines and avid in vivo uptake in mice xenografted with HER2-positive tissues. The authors also enrolled two human patients with primary breast cancer for an initial in-human investigation of ^68^Ga-NOTA-MAL-Cys-MZHER_2:342_ PET imaging, one with HER2-positive disease and another with HER2-negative disease. The former patient had significantly more avid uptake of the radiotracer. In addition, the low background radioactivity observed with this radiotracer may prove useful for detecting metastatic lesions in the future, particularly those in the liver [111].

### 5.7. ^18^F-Labeled 1-Amino-3-Fluorocyclobutane-1-Carboxylic Acid (^18^F-Fluciclovine)

The positron emitter ^18^F-labeled 1-amino-3-fluorocyclobutane-1-carboxylic acid (^18^F-fluciclovine) is being investigated as an imaging agent for various cancers. This radiotracer has been FDA-approved for use in PET imaging of men with suspected recurrence of prostate cancer after initial treatment [112]. Initial trials in breast cancer, particularly ILC, have shown promising results, suggesting that ^18^F-fluciclovine may be more effective than ^18^F-FDG in diagnosing ILC. Although the number of ILC patients studied so far has been small, preliminary evidence indicates that changes in the corrected SUV_max_ of ^18^F-fluciclovine PET/CT are associated with ILC tumor response [113].

### 5.8. 3′-Deoxy-3′-[^18^F]-Fluorothymidine (^18^F-FLT) and ^18^F-DPA-714

^18^F-FLT is an imaging agent that indicates the level of cell proliferation. Ma et al. conducted a preclinical study that was the first trial to employ ^18^F-FLT PET/CT to forecast early responses to CDK4/6 inhibitors in TNBC [114]. The study found that ^18^F-FLT PET/CT can successfully track early treatment responses shown by a significant decrease in ^18^F-FLT uptake and tumor volume in MDA-MB-231 cells after therapy. In addition to that, ^18^F-DPA-714 is a second-generation translocator protein 18 kDa (TSPO) tracer that is also being investigated in TNBC. TSPO is a sensitive macrophage marker with potential applications in TNBC classification. A preliminary study showed ^18^F-DPA-714 uptake with different kinetic patterns potentially linked to TSPO polymorphism status [115].

### 5.9. Others

Other tracers have also been studied in breast cancer. They are less commonly used and more studies are needed to evaluate their potential role in tumor detection. Such tracers include ^68^Ga-PSMA-HBED-CC, 16β-[^18^F]fluoro-5α-dihydrotestosterone (^18^F-FDHT), and ^68^Ga-RM2, which have been studied primarily in prostate cancer [116,117,118], as well as ^68^Ga-Pentixafor, which has been studied primarily in hematological malignancies [34].

## 6. Future Directions

Theranostics is an emerging field that integrates diagnostic imaging and therapeutic interventions and has become a promising approach to personalized medicine, especially in the treatment of cancer. In regard to nuclear imaging of breast cancer, theranostics may involve the use of radiolabeled therapeutic molecules such as monoclonal antibodies and peptides. Numerous targeted radionuclide therapies have shown potential, including for example ^177^Lu-trastuzumab for HER2-positive breast cancer, and ^90^Y-FAPI-04 and ^177^Lu-FAPI-46 for advanced stage malignancies and metastatic breast cancer [119]. In addition, new techniques that incorporate nanotechnology have emerged and have proven particularly effective in TNBC [120]. Several trials and phase 1 or phase 2 studies are also ongoing for the development of new radionuclide-based treatments for breast cancer [121]. Although the treatment of breast cancer has progressed significantly in recent years, many patients still fail the currently available therapeutic modalities and are left with minimal options. Theranostic approaches represent a potential option for such patients in the future and have the potential to greatly improve the diagnosis, treatment, and outcomes of breast cancer. However, limited evidence is available and primarily focuses on compassionate use for pain management and palliative therapy rather than treatment. Further studies are needed to investigate new molecules and applications and expand this promising field [119].

## 7. Conclusions

In conclusion, radiotracers have significantly enhanced breast cancer imaging, with current techniques like PET using tracers such as ^18^F-FDG and FAPI proving invaluable in clinical practice. These methods improve tumor detection, characterization, and treatment monitoring. Future advancements, including novel tracers targeting specific molecular pathways like HER2 and ER, hybrid imaging technologies such as PET/MRI, and radionuclide-based treatments, promise even greater precision and personalization in breast cancer care. Ongoing research and clinical trials are crucial to validate these innovations, which hold the potential to further improve early detection, treatment planning, and patient outcomes.

## Data Availability

No new data were created or analyzed in this study. Data sharing does not apply to this article.
